# “Negative Energy Magnetic Field”: A Descriptive Qualitative Study on Occupational Stressors among Chinese Hospice Nurses

**DOI:** 10.1155/2024/3311735

**Published:** 2024-08-29

**Authors:** Yanming Wu, Ya Mao, Yangchenchen Liu, Erming Yang, Yuxin Zhou, Yuanyuan Jin, Hui Yang

**Affiliations:** ^1^ Nursing College of Shanxi Medical University, Taiyuan, Shanxi, China; ^2^ Department of Psychology University of Minnesota, Minneapolis, MN, USA; ^3^ Cicely Saunders Institute of Palliative Care, Policy and Rehabilitation Florence Nightingale Faculty of Nursing Midwifery & Palliative Care King's College London, London, UK; ^4^ School of Nursing Medical College of Soochow University, Suzhou, Jiangsu, China; ^5^ First Hospital of Shanxi Medical University, Taiyuan, Shanxi, China

## Abstract

**Methods:**

A descriptive qualitative approach was used. We conducted semistructured interviews with 30 hospice nurses from 14 cities in China between August 2023 and February 2024. Data were analyzed using conventional content analysis. The study adhered to the COREQ checklist for reporting.

**Results:**

Hospice nurses perceived themselves as immersed in a persistent “negative energy magnetic field,” emphasizing the pervasive stress they experienced in their work. There are four different levels of occupational stressors among Chinese hospice nurses: (1) individual-level stressors such as difficulty in managing physical symptoms, dealing with futile resuscitations, and struggling with emotional boundaries; (2) organizational-level stressors encompassing insufficient financial support and human resources, negative leadership behaviors, and conflicting philosophies in healthcare; (3) societal-level stressors involving challenges such as avoidance of conversations about death, pragmatism, and implicit communication modes; and (4) acute stressors including patient suicide and sudden patient death*. Conclusions*. Diverse occupational stressors faced by hospice nurses are greatly influenced by culture. Future research should thoroughly examine these stressors at various levels and consider the cultural impacts on the stress experienced by hospice care nurses within a broader context.

## 1. Introduction

In China, the population aged 60 and above reached 280 million by the end of 2022, constituting 19.8% of the total population [1]. Meanwhile, the incidence of new cases of malignant tumors was 4.064 million, with a rate of 293.91 per 100,000 in 2016 [2]. As the elderly population grows rapidly and the number of cancer patients continues to increase, the demand for hospice care in China is rising. The government has shown considerable interest in advancing hospice care, as evidenced by the issuance of the “Healthy China 2030 Outline,” which explicitly emphasizes the necessity of bolstering the infrastructure of hospice care to ensure comprehensive healthcare services spanning from prenatal care to end-of-life care [3]. To achieve this goal, the Chinese government launched the first batch of nationwide pilot work on hospice care in five cities in 2017 and subsequently increased the number of pilot cities twice in 2019 and 2023. Despite increased government support for hospice care in recent years, the development of hospice care in China is still in its infancy. This is mainly due to insufficient education about hospice care, inadequate staffing of hospice care teams, and uneven distribution across regions [4, 5].

Caring for terminal patients creates a challenging and high-pressure work environment for hospice nurses. Terminal patients seek more care, comfort, and emotional support, and hospice nurses play a crucial role in providing these aspects. Hospice care aims to relieve patients' physical symptoms and extends its responsibility to address patients' psychological, mental, social, and spiritual needs [6]. The expectations of meeting diverse needs pose higher demands for hospice nurses. They must not only care for patients experiencing pain and other symptoms but also help them navigate the uncertainty surrounding their death [7]. Additionally, they are expected to fulfill families' various needs while facing potential risks in the process of caregiving, such as providing grief counseling for bereaved family members [8].

Occupational stress is an individual's subjective experience of stressors resulting from the imbalance and mismatch between work demands and response capacities [9]. Previous studies found that excessive occupational stress can have negative impacts on nurses' physical health and emotions during their work, leading to reduced productivity, compromised the quality of nursing care. Excessive occupational stress among nurses can lead to detrimental outcomes for all parties involved: nurses, patients, and hospitals [10–12]. Quantitative studies from Germany [13, 14] and Japan [12] reported that hospice nurses experienced a medium-to-high level of work-related stress, primarily due to main occupational stressors such as high workload, high emotional demands, and chronic exposure to death. One study on hospice nurses in the United States showed that the most significant occupational stressor among nurses was assisting patients and their families in coping with death, followed by workload issues, such as a shortage of human resources and insufficient time to provide psychological support to patients [15]. Additionally, relationships with patients, families, and team members were identified as both stressors and sources of energy for hospice nurses, depending on how they were perceived and managed [16]. Therefore, recognizing hospice nurses' stressors at an early stage and assisting them in effectively coping with and transforming stressors is critically important to improve nurses' psychological health.

Most studies on occupational stressors among hospice nurses have applied quantitative research methods. While quantitative research is helpful for identifying stressors using structured assessment measures, it may overlook significant stressors from nurses' perspectives and fail to consider cultural influences on these stressors [17]. Qualitative research serves to address this deficiency by understanding hospice nurses' occupational stressors from their own perspectives and experiences, aiding in the identification of previously neglected stressors [18]. Compared to Western countries, hospice care in China is in its early developmental stage, and hospice nurses may face specific stressors during this phase [19]. Additionally, stressors are affected by cultural backgrounds [20]. Different countries and regions have different perspectives on dying, death, and the afterlife due to their diverse cultural backgrounds, making hospice care highly culturally diverse. The psychological, spiritual, and social needs of terminal patients vary widely in different regions. The traditional Chinese worldview of life and death has a profound influence on patients' acceptance of hospice care, their decisions about end-of-life treatment and care, and the way they express their personal care needs, which may affect the occupational stress of hospice nurses to some degree [21]. Specifically, the Chinese death culture is characterized by a “reverence for life and aversion to death,” where death is seldom discussed openly as it is still considered taboo and inauspicious [22]. Most Chinese people tend to pursue longevity, rather than the quality of life. This cultural perspective on death causes terminal patients and their families to fear death, pursue treatment at all costs, and hold a negative attitude toward hospice care, viewing it as merely waiting for death [23]. This, in turn, leads to resistance against the care provided by hospice nurses, causing them considerable stress in their work.

To date, no studies to the authors' knowledge have comprehensively examined the occupational stressors experienced by hospice nurses in China. Existing literature has mostly discussed job burnout and compassion fatigue among hospice nurses [24–26]. Deepening our contextual knowledge of this area is crucial for cultivating supportive work environments to manage stress effectively. Therefore, this study aims to investigate the occupational stressors experienced by hospice nurses in China, contribute additional evidence on this topic for other countries, and provide insights for stress management among hospice nurses in culturally similar regions. Additionally, the findings of this qualitative study can lay the groundwork for developing specific stress screening tools and establishing coping strategies.

## 2. Methods

### 2.1. Design

A qualitative descriptive approach is beneficial for collecting detailed, direct, and firsthand descriptions of a phenomenon with limited available information [27]. This design is suitable for discovering and understanding a phenomenon, a process, or the perspectives and worldviews of individuals involved in research [28]. Therefore, in this study, this approach was selected to gain a comprehensive understanding of hospice nurses' perceptions regarding occupational stressors. This study was reported following the Consolidated Criteria for Reporting Qualitative Research (COREQ) checklist.

### 2.2. Settings, Recruitment, and Sampling

Participants for this study were recruited through invitations sent to institutions and at conferences. For institutional recruitment, we purposively sampled hospice care institutions in Taiyuan, Suzhou, and Changsha. Prior to entering these institutions, we sent a “Research Opportunities Inquiry at Your Institution” (Supplementary Material 1) to seek permission from administrators. Administrators then introduced the study to hospice nurses and recruited potential participants. For conference recruitment, the interviewer (YW) attended the National Hospice Care Nursing Advances Seminar hosted by the Chinese Nursing Association in July 2023 and the Guangdong Province Hospice Care Service Skills Training Workshop in October 2023. At these conferences, the interviewer actively invited hospice nurses across China to participate in the study. Following these methods, interested participants were contacted and provided with detailed study information. They were given at least 24 hours to consider whether to participate in the study. Given the relatively limited number of hospice nurses in China and their dispersed distribution, snowball sampling was employed after each interview to invite qualified acquaintances to join the study, aiming to gather more comprehensive data [29]. Furthermore, small gifts were given to the research participants to acknowledge the time and effort they have provided in participating in the research.

### 2.3. Participants

From August 2023 to February 2024, researchers recruited hospice nurses from fourteen medical institutions nationwide. The inclusion criteria were: (1) having a hospice nursing qualification certificate and possessing more than one year of hospice care experience and (2) consenting to participate in the current study. The exclusion criteria were: (1) nurses who were not in the period of rotation of hospice care or had left this work and (2) standardized training nurses, refresher nurses, and nurse interns. The sample size was determined by the principle of data saturation, ensuring that no new emerging information was obtained [30].

### 2.4. Data Collection

The interviews were conducted by YW, a female doctoral student in nursing with training in qualitative interviewing, psychological counseling, and specialized hospice nursing. YW completed her master's degree in nursing in Suzhou and is currently pursuing her doctoral degree in nursing in Taiyuan. She audited a two-week specialized hospice nurse training course by the Chinese Nursing Association in Changsha. This background helped her quickly build rapport with participants and grasp cultural cues and dynamics during interviews. After piloting interviews with two hospice nurses and refining the interview guide, the final version covered two main areas: (i) general experiences in end-of-life care and (ii) stressful situations faced by hospice nurses at work. During each interview, further flexible probing and exploration were conducted based on the participants' responses. YW explained the interview purpose and procedures, obtained consent, and scheduled meeting times. Interviews were conducted in a quiet, safe, and private setting. Considering the dispersed nature of hospice nurses and limited financial and human resources, the study utilized both face-to-face and telephone interviews to enhance response rates [31]. All interviews were recorded, and reflexive field notes were taken to capture the researcher's reflections for subsequent interviews. After 27 interviews, data saturation was achieved, with an additional three interviews conducted for confirmation [32].

### 2.5. Data Analysis

Interviews were recorded and transcribed verbatim within 24 hours. The current study used conventional content analysis to analyze interview data [33]. This method takes a data-driven “bottom-up” approach to analyze data without specific research hypotheses. Instead, it allows researchers to actively identify codes, subcategories, and categories based on participants' responses. Specifically, the first and second authors independently delved into the interview data, repeatedly reading text files and immersing themselves in the context to gain a general sense of the data. Next, they read the text data word by word, condensed, and extracted meaningful units related to stressors of hospice nurses from the text, and then began coding. After the number of codes was saturated, the two authors organized the codes into subcategories and categories based on the connections among code attributes and codes. Lastly, they defined each category and subcategory. To ensure the consistency of the analysis, the research team discussed regularly and reached a consensus on codes, subcategories, and categories throughout the data analysis process.

### 2.6. Rigor

Guba and Lincoln's principles were adopted to ensure the rigor and trustworthiness of this study [34]. First, this study conducted interviews with hospice nurses from fourteen cities across east, south, west, north, and central China, representing wide-ranging age groups, various lengths of work experiences, different educational backgrounds, and varied professional titles to enhance the diversity of perspectives. Then, two researchers independently coded the data and regularly reported the results to the research group. This approach aimed to avoid the influence of researchers' presumptions on the analysis process and to enhance the credibility of the results. Finally, the first author conducted member-checking, returning research data to participants to verify the accuracy and alignment of the study results with their experiences.

### 2.7. Ethical Considerations

This study obtained ethical approval from the Ethics Committee of The First Hospital of Shanxi Medical University (Ref: NO. KYLL-2023-021) and the Shanxi Medical University Research Ethics Committee (Ref: 2024002). The researchers explained the purpose of study to the participants and received their consents before interviews. Written informed consent was obtained from all participants prior to the interview. Participants' identifiable information, including personal names and employer names, has been de-identified. All data were collected solely for research purposes and kept confidential and undisclosed to the public.

## 3. Results

Thirty hospice nurses were interviewed from fourteen cities across China. Researchers conducted in-person interviews (*n* = 15) with hospice nurses from Taiyuan, Suzhou, and Changsha. In addition, virtual interviews (*n* = 15) were conducted with nurses from the remaining cities due to personnel and financial reasons. Each interview took about 45 to 60 minutes. More details on the final participant characteristics are shown in Table 1.

**Figure 1:**
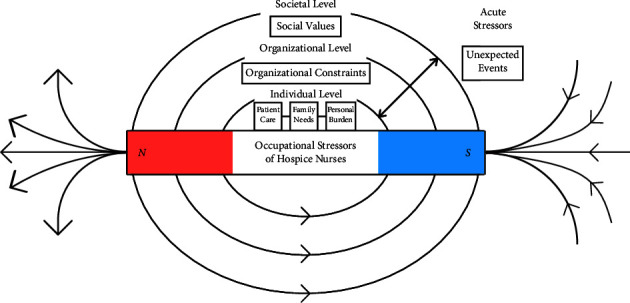
The “negative energy field” model of hospice nurse stressors.

The “Negative Energy Field” model of hospice nurse stressors (Figure 1) draws on the structure of the “magnetic field” and the concept of “systems thinking.” Systems thinking argue that the only way to fully understand one thing is to understand the parts in relation to the whole [35]. Hospice nurses described feeling surrounded by various stressors in end-of-life care, creating a pervasive sense of being in “negative energy field” (e.g., “This is where many life pains converge, and stress permeates everything.”). Hospice nurses' perceptions of occupational stressors were categorized into four types: individual-level stressors, organizational-level stressors, societal-level stressors, and acute stressors (see Figure 1). It is worth emphasizing that acute stressors may affect hospice nurses across individual, organizational, and societal levels. Therefore, based on the study findings and researcher reflection, acute stressors are depicted spanning each level in Figure 1. More details on categories, subcategories, and quotes can be found in Table 2.

### 3.1. Individual Level

#### 3.1.1. Patient Care Difficulties

Terminal patients, who are the primary recipients of hospice care, directly influence hospice nurses through their suffering, leading to distress among the nurses. Specifically, the inability to relieve patients' physical discomforts and to deal with patients' complex and ever-changing mental states has contributed to significant stress for hospice nurses.

*(1) Difficulty in Managing Physical Symptoms*. Participants reported that symptoms of terminal patients were complex, and the final stages of diseases are often the most challenging to manage. This not only imposes a significant workload on nurses but also causes substantial stress when patients' symptoms cannot be effectively relieved.

*“Their [the patients'] wish in coming here is to be comfortable. They want to experience comfort during the last stage of life. Patient families also desire comfort, wishing for no pain, no suffering, and no depression. The most significant stress for us is that we sometimes struggle to manage patients' symptoms despite our best efforts.”* (N6).

*(2) Complex and Ever-Changing Mental States*. Mental states of terminal patients are constantly in an unstable condition due to the deterioration of illness and the approach of death. Hospice nurses reported feeling a high level of stress when facing such situations.

*“The patients we work closely with are terminal patients. They may have a very bad temper and could suddenly curse or lose their temper while you are providing them with care. They also show negative attitudes toward you. It is not because of your care quality but because they need a place to vent their emotions.”* (N8).

*(3) Lack of Standards in Spiritual Care*. Spiritual care is a significant component in hospice care. It is personalized, unstandardized, and requires specific techniques. Moreover, the spiritual needs of terminal patients differ from those of ordinary patients. In addition to enduring physical pain, terminal patients also confront the inevitable risk of death. Many participants expressed that spiritual care is often the most stressful aspect of hospice care.

*“Individuals have varied spiritual needs, so there is no single correct answer or set procedures guiding you in providing spiritual care. It [spiritual care] changes all the time. Therefore, it's quite stressful.”* (N5).

#### 3.1.2. Family Needs

In Chinese culture, the opinions and attitudes of the family are highly valued, particularly when making critical decisions. Therefore, hospice nurses must consider the feelings of the family when inquiring about patients' preferences and discussing patients' conditions and death. Nurses are often asked not to disclose patients' true diagnosis and prognosis until consent is obtained from their families. This situation has made their work challenging, as the nurses are expected to consider the family's needs while providing hospice care according to the patients' preferences.

*(1) Futile Resuscitations*. Under the influence of China's traditional culture of familism, families might request medical staff to actively resuscitate and treat patients when faced with the impending death of terminal patients. The unnecessary resuscitations conflict with the principles of hospice care, which involve allowing patients to pass away with dignity, without pain, and the decision to forgo futile resuscitations. Therefore, hospice nurses often experience confusion and stress due to these futile resuscitations.

*“Not every patient in our units can leave peacefully because some families persistently ask for resuscitations, possibly influenced by China's traditional values. These families believe that they must resuscitate their parents to show filial piety and care. I find it painful to witness these resuscitations for terminal patients, but we must comply in most situations. That's what the family asks for, and we must follow.”* (N1).

*(2) Disease Concealment*. The families of patients may request that nurses withhold information about the patients' actual medical conditions from the patients, seeking to prevent psychological shocks arising from awareness of their illnesses. Concealing these illness conditions has placed additional stress and challenges on the daily care duties of nurses.

*“I believe that every patient family who conceals illness conditions from the patients will eventually regret it. While I would suggest not concealing such information, I respect the family's decisions, and I have no right to interfere. Therefore, I will be more careful in handling these situations and feel stressed in the meantime.”* (N8).

#### 3.1.3. Personal Burdens

Hospice nurses can experience stressors related to personal factors at work, including worries about insufficient professional knowledge and skills to achieve the goals of hospice care, difficulties in managing nurse-patient relationships, and anxiety and fear about death due to prolonged exposure to patients' deaths.

*(1) Lack of Knowledge and Skills*. Some hospice nurses believed that they lacked professional knowledge and skills, and they worried that they were not competent enough to provide high-quality care services to patients.

*“There are situations where I find myself unsure of how to handle them, possibly due to a lack of capabilities and techniques. I feel that I haven't learned enough or don't possess sufficient knowledge, and this can be quite stressful.”* (N5).

*(2) Struggle of Emotional Boundaries*. Because of the challenges in controlling the level of emotional involvement with patients, hospice nurses frequently encounter difficulties in handling their emotions after patients pass away, resulting in adverse emotional outcomes.

*“I think that our job is kind of cruel. You need to build emotional relationships with patients, but every relationship has the same outcome—they will pass away. Therefore, I think it's very cruel and very sad for us, hospice nurses. This requires a strong psychological state. Every time I think of their leaves, I feel deeply sorrowful.”* (N11).

*(3) Death Anxiety*. The hospice nurses reported experiencing anxiety related to death and questioning the meaning of existence, after repeatedly facing the shocks of patient deaths.

*“I believe that working in the hospice unit can be challenging because, at times, emotions are difficult to control. After witnessing patients' deaths, I occasionally find myself losing hope in life and feeling that things are meaningless.”* (N14).

### 3.2. Organizational Level

#### 3.2.1. Organizational Constraints

In a team of collaborative members with diverse personalities and varied professional backgrounds, differences in opinions on patient treatment or care arise, and consensus communication is lacking. Hospice nurses also reported stressors stemming from the lack of resources and support for nursing work from management, including negative leadership behaviors.

*(1) Insufficient Financial Support and Human Resources*. Participants expressed that the current state of hospice care and the policy environment in China are still in their infancy. The funding and human resources for conducting hospice care are insufficient, resulting in a lack of adequate financial support for nursing work. Hospice nurses invest a significant amount of time and effort but receive comparatively minimal remuneration, which becomes a stressor for them.

*“Personnel are very lacking. Many hospice nurse specialists are not specially providing hospice care. Most of them must take on responsibilities in other wards and provide hospice care during their free time. So, this is very challenging. In fact, all our cases involving spending our own time and money to care for patients.”* (N8).

*(2) Negative Leadership Behaviors*. Many participants indicated that head nurses, as pillars for nursing staff, exhibit negative leadership behaviors during the management process. For example, scolding nurses in public and adopting an unsupportive attitude toward their work, which are significant stressors for the nurses.

*“The head nurse frequently criticizes and scold us in the morning meetings. Working in a department where death is a common occurrence is already quite stressful. Having to face criticism early in the morning adds to the stress throughout the day. I find this situation very frustrating.” (N2).*

*(3) Conflict Philosophies in Healthcare*. Participants expressed that, at the current stage, China is actively cultivating specialized hospice nurses but lacks a focused training program for hospice specialized doctors. This has led to a certain level of mismatch in the medical and nursing philosophies in actual work. Many doctors have not provided sufficient support for hospice care, creating obstacles in the practice of hospice nursing care.

*“Nurses have a higher passion for hospice care. But as a nurse, your voice is often not heard. After all, doctors have all the rights to decide how to treat patients, and they are seen as having a higher status by patients. Nurses face many stresses during their practices, especially when doctors don't recognize your work.”* (N19).

### 3.3. Societal Level

#### 3.3.1. Social Values

China's traditional culture, with Confucianism as the principal part, has shaped the values, beliefs, customs, and behaviors of Chinese people. It also has a profound influence on people's attitudes towards hospice care.

*(1) Avoid Conversations about Death*. Chinese culture emphasizes the idea of “emphasizing birth and downplaying death.” Specifically, people rarely directly talk about death and even view death as a taboo and an inauspicious topic. This perspective has led to misunderstandings and rejections toward hospice nurses and their work, causing stress for the nurses.

*“When we tried to promote hospice care to the patient, they did not accept it very well due to the impact of our Chinese traditional cultures. They would say that death bring bad luck, then they always avoid any conversations about it. People always misunderstand about hospice care, and this misunderstanding make you feel stressful.”* (N23).

*(2) Pragmatism*. The Chinese style pragmatism emphasizes the most direct application and short-term benefits. Some terminal patients considered certain comforting care in hospice care as lacking practicality for treating illness. Therefore, they often express attitudes of skepticism and reluctance, and it has created a certain amount of stress for hospice nurses.

*“We provide some comforting services to the patients, such as meditation and aromatherapy, but they perceive these as ineffective for their illness. You meticulously prepare those services, but it doesn't come with positive feedback, then you would feel stressed. I even started to question the purpose of this job.”* (N6).

*(3) Implicit Communication Mode*. In Chinese culture, people usually tend to follow the principle of restraint. They adopt implicit and veiled ways of expressing thoughts and emotions, further forming a culture that values reserved emotions. In hospice care, patients and their families often have ambiguity and flexibility in their expressions, presenting a challenge in nurse-patient communication. Nurses often find it difficult to accurately discern the genuine needs of patients and their families.

*“Chinese people are often reserved, and many of them may not be adept at expressing themselves or do so in an ambiguous way. So, we can't have some deep conversations, and this could be another stress in my work.”* (N21).

### 3.4. Acute Stressors

#### 3.4.1. Unexpected Events

When unexpected events occur, such as patient suicide or sudden patient death, nurses may experience acute stress across individual, organizational, and societal perspectives in the short term. Specifically, these events may result in blame from the patient's family and self-doubt at the individual level; questioning from nursing managers or doctors about their patient monitoring abilities at the organizational level; and undermining the perceived value and acceptance of hospice nursing at the societal level.

*(1) Patient Suicide*. Hospice nurses have the mission of facilitating a peaceful death for patients. When confronted with situations where patients choose suicide to end their pain and suffering, hospice nurses are profoundly shocked, experiencing emotions such as anger, fear, and self-blame. Society also questions the professionalism of nurses.

*“We have a patient committed suicide during the night, without a single sign. You can't figure out why this happened. After that, I become very concerned about the patients, especially about their psychological problems.”* (N6).

*(2) Sudden Patient Death*. Terminal patients may experience sudden deaths due to the severity of their illnesses. Families of the patients often struggle to accept these sudden deaths. Additionally, doctors and nursing supervisors may attribute them to the nurses' dereliction of duty. Hospice nurses, in turn, express feelings of self-doubt and self-blame regarding the sudden deaths of patients.

*“Patients could pass away peacefully in their sleep, with stopped heartbeats and no breaths, even if they were not in a severe condition. When patient families touched their bodies, they found them cold. Overwhelmed with grief, they rushed to the nurse station, crying and eventually breaking down. You can imagine how stressful it was for us*.*”* (N16).

## 4. Discussion

In this study, we employed a descriptive qualitative approach to directly investigate hospice nurses' occupational stressors in their own words. Additionally, we used conventional content analysis, a data-driven “bottom-up” approach, to capture insights directly from participants. This method mitigates researcher bias and provides novel perspectives on the data. This study delves deeply into the occupational stressors from the perspective of hospice nurses. They perceived themselves as being immersed in a persistent “negative energy magnetic field,” highlighting a pervasive sense of being engulfed by layers of stress. Within this field, they not only encounter various stressors but are also surrounded by an insidious energy that casts a pall over the entire work environment. This depiction underscores the ubiquity of stress and conveys the complex and profound challenges that nurses face in their work. The field itself emits a vibe of “Like charges repel each other,” further emphasizing the intensity of the stress and its inescapable nature within the work setting.

The study found similar results to previous studies, indicating that the inability to alleviate patients' physical symptoms [36], the complex and variable psychological states of terminally ill patients [37], the inability to adequately meet patients' spiritual needs [38], and a lack of self-knowledge and skills [39] can contribute to a certain level of psychological stress for nurses. Furthermore, our findings indicated that the absence of emotional boundaries in nurses is also a major source of stress. Hospice care is a practice that involves both professionalism and emotion, making it difficult for nurses to entirely separate professional relationships from personal ones, especially within the context of relationally oriented Chinese culture [40]. The lack of emotional boundaries during patient care causes significant emotional distress (e.g., compassion fatigue and vicarious trauma) for hospice nurses [41, 42]. Establishing emotional boundaries is a key strategy in mitigating emotional distress. Although challenging in clinical practice, there are methods to maintain these boundaries. For instance, hospice nurses can prevent and alleviate emotional distress through self-awareness and mindfulness techniques [43]. Organizations should enhance emotional training, regularly assess mental health, and provide professional psychological support when needed [44]. The current literature offers limited practical techniques for hospice nurses to maintain emotional boundaries, indicating a need for future research in this area.

It is worth noting that some special requests from patients' families, such as futile resuscitation and concealment of the condition, also often put nurses under great pressure. Influenced by Chinese traditional familism, family decisions often take precedence over the wishes of terminally ill patients themselves. In addition, due to the traditional filial piety values in China, making every effort to save lives is generally regarded as the epitome of “filial piety” [45]. Consequently, family members often request medical personnel to actively resuscitate and treat patients who are on the brink of death. This often leads to overtreatment of terminally ill patients, in particular the use of cardiopulmonary resuscitation. This conflicts with the hospice care principle of allowing patients to die with dignity and without pain, and abandoning end-of-life resuscitation, which also causes stress for hospice nurses. The second stressor from patients' families is concealing the medical condition. Due to China's cultural background, families often choose to conceal a patient's terminal condition. [46]. It is assumed that disclosing the true condition aggravates the patient's condition and leads to tension and anxiety. Whether maintaining confidentiality regarding the illness or disclosing it to the patient presents a common source of stress and dilemma for hospice nurses. In China, families play central roles in hospice. Consequently, initiatives such as life and death education for the public should be implemented to gradually raise awareness of hospice care throughout society.

From an organizational perspective, insufficient funding and human resources are also stressors, consistent with the findings of Ling's research [4]. Furthermore, our findings showed that negative leadership behaviors have adverse effects on hospice nurses' emotions and motivations. The Chinese social context is characterized by collectivism and influenced by traditional Confucian thought, with deep-rooted concepts of hierarchical respect, causing significant distress to nurses due to negative leadership behaviors [47]. Additionally, conflicts between medical and nursing philosophies are also a prominent source of stress for hospice nurses. In the development of hospice care in China, nursing has taken the lead over medicine, with the nursing profession placing a stronger emphasis on the philosophy of hospice care. For instance, since 2019, the Chinese Nursing Association has been conducting training for hospice care specialty nurses, whereas there is currently no specialized training for hospice care doctors in China. The nursing profession also started offering degrees and graduate education in hospice care studies in 2024 [48]. Overall, hospice care competency training for doctors in China is insufficient. However, in clinical practice, doctors usually assume a leading role, leading to patient identification and trust in doctors' decisions. Conflicts between medical and nursing philosophies in such situations create stress for nurses.

From the social perspective, former studies emphasized how the public's fear of death puts pressure on hospice care nursing in China [39]. Within the Chinese cultural background, our study is the first to identify that pragmatism and implicit communication modes impact on the work of hospice nurses. Firstly, unlike the philosophical concept of pragmatism, Chinese pragmatism places greater emphasis on “practicality” and stresses “immediate effectiveness” [49]. Some humanistic practices in hospice care, which patients believe are of no substantive or immediate benefit to their illness, often lead to questioning and reluctance to accept, which to some extent also causes distress for nurses. Furthermore, the lack of freedom of thought and expression formed by China's long-standing feudal autocracy and constraints from Confucianism deeply affects all aspects of Chinese society. “Harmony” is the core of the Confucian system of benevolence and a significant aspect of Chinese philosophy of life [50]. The concept of “harmony” dictates subtlety and indirectness in language expression. Chinese people often exercise restraint in expressing thoughts and emotions, forming a national psyche that values subtlety and implications. In hospice care, the subtlety and flexibility exhibited by patients and family members create communication pressures between nurses and patients, as nurses often struggle to identify the true needs of patients and family members.

Our findings also revealed that sudden events such as the unexpected death or suicide of patients represent a particularly acute source of stress for hospice nurses. The sudden death of a patient due to rapidly worsening conditions can be shocking for families. Doctors and nursing managers may consider whether nurses' rounds and observations of the condition were timely and appropriate. The societal perception of nurses' professional identity is compromised. Hospice nurses may consequently experience doubt and self-blame, subsequently facing significant psychological pressure from various sources in the short term. Moreover, hospice care strives to assist terminal patients in experiencing a peaceful end of life. However, when patients end their lives through suicide, it shakes the belief systems and sense of mission of hospice nurses, leaving them feeling pressured and apprehensive. The American Association of Suicidology has coined the term “Clinician Survivors of Suicide Loss,” referring to professional caregivers who experience the suicide of their own patients [51]. Clinician survivors bear the dual roles of caregiver and bereaved, thereby placing tremendous pressure on them, such as undergoing self-review or feeling defeated. We recommend that clinics should improve emergency management in response to sudden incidents in hospice care and pay close attention to the psychological state of hospice nurses, offering proactive support as needed.

## 5. Limitation

Due to practical constraints, this study focused exclusively on female hospice nurses, thereby limiting the exploration of gender differences in stress perceptions and experiences. However, we ensured diversity in the interview sample by including nurses of various ages, work experience levels, educational backgrounds, professional titles, and geographic regions. Additionally, this study was conducted within the unique cultural context of China, which may limit the transferability of the findings to other distinct cultural settings.

## 6. Strengths and Implication

Despite these limitations, our study has several strengths compared to previous studies. First, culturally relevant stressors found in this study may be applicable to countries with similar cultural backgrounds. Hospice care is deeply connected to local death culture. It is also necessary to consider cultural influences when exploring stressors among hospice nurses across different countries. Second, the results of this qualitative study can serve as a foundation for creating targeted stress screening instruments and formulating effective coping mechanisms. Lastly, the study highlights that hospice nurses experience stressors such as unmet psychological or spiritual patient needs and conflicts in healthcare philosophies. This underscores the importance of proactively enhancing interdisciplinary collaboration within the hospice care team, comprising physicians, nurses, psychologists, social workers, and others, to better address the diverse needs of terminally ill patients. These efforts will promote a healthier work environment for hospice teams, benefiting terminal patients and the healthcare system as a whole.

## 7. Conclusion

This study provides a detailed understanding of occupational stressors among hospice nurses in China, which fills the gaps identified in previous research. The findings also describe how diverse and interconnected stressors may complicate their end-of-life care duties. Our evidence-based recommendations can inform the development and implementation of targeted strategies to cope with these stressors in hospice nurses across China and similar cultural contexts in the Asia-Pacific region. Future research should focus on fully understanding occupational stressors by examining stressors at various levels and considering the influence of culture on the stress experienced by hospice care nurses within the broader context.

## Figures and Tables

**Table 1:** Characteristics of the participants (*N* = 30).

Nurses	Gender	Age	Education	Title	Hospice care experience (year)
N1	Female	46	Bachelor's degree	Supervisor nurse	15
N2	Female	37	Bachelor's degree	Associate professor of nursing	3
N3	Female	31	Bachelor's degree	Supervisor nurse	1
N4	Female	35	Bachelor's degree	Supervisor nurse	2
N5	Female	37	Bachelor's degree	Supervisor nurse	1
N6	Female	36	Bachelor's degree	Supervisor nurse	5
N7	Female	37	Bachelor's degree	Supervisor nurse	4
N8	Female	35	Bachelor's degree	Supervisor nurse	1
N9	Female	36	Bachelor's degree	Supervisor nurse	6
N10	Female	33	Bachelor's degree	Supervisor nurse	5
N11	Female	25	Bachelor's degree	Senior nurse	3
N12	Female	33	Bachelor's degree	Supervisor nurse	4
N13	Female	34	Bachelor's degree	Supervisor nurse	4
N14	Female	47	Associate's degree (3 years)	Supervisor nurse	8
N15	Female	38	Bachelor's degree	Senior nurse	7
N16	Female	25	Associate's degree (3 years)	Senior nurse	5
N17	Female	30	Master's degree	Senior nurse	2
N18	Female	33	Bachelor's degree	Supervisor nurse	1
N19	Female	41	Bachelor's degree	Supervisor nurse	2
N20	Female	25	Bachelor's degree	Senior nurse	3
N21	Female	29	Associate's degree (3 years)	Senior nurse	7
N22	Female	26	Bachelor's degree	Senior nurse	4
N23	Female	36	Bachelor's degree	Supervisor nurse	4
N24	Female	33	Bachelor's degree	Supervisor nurse	3
N25	Female	40	Master's degree	Associate professor of nursing	5
N26	Female	25	Bachelor's degree	Nurse	1
N27	Female	27	Master's degree	Supervisor nurse	1
N28	Female	29	Master's degree	Senior nurse	2
N29	Female	39	Bachelor's degree	Supervisor nurse	5
N30	Female	32	Bachelor's degree	Supervisor nurse	4

**Table 2:** Categories, subcategories, and exemplar quotations.

	Categories	Subcategories	Quotations
Individual level	Patient care difficulties	Difficulty in managing physical symptoms	Their [the patients'] wish in coming here is to be comfortable. They want to experience comfort during the last stage of life. Patient families also desire comfort, wishing for no pain, no suffering, and no depression. The most significant stress for us is that we sometimes struggle to manage patients' symptoms despite our best efforts. (N6) Patients have questioned me, “I am here to reduce symptoms. Why are there no solutions? Why can't you do it? What are you doing every day? I don't want to stay here anymore. Do you have any pills to poison me to death?” These questions are constantly shocking. Some patients even say, “Let me die,” repeat this over and over. At that moment, you truly feel that the stress has reached its peak. (N10) You watch this situation hopelessly. You want to intervene, to alleviate some symptoms, but you are powerless. (N14)
		Complex and ever-changing mental states	The patients we work closely with are terminal patients. They may have a very bad temper and could suddenly curse or lose their temper while you are providing them with care. They also show negative attitudes toward you. It is not because of your care quality but because they need a place to vent their emotions. (N8) The emotions of terminal patients are very unstable. You can easily sense their feelings of upset or depression. I find it challenging to talk with them. I don't know how to relieve their emotions, how to identify their problems, or how to assist them in expressing or releasing repressed emotions. (N12) The ever-changing emotions, symptom discomforts, and the lack of safety among the patients all contribute to bringing me stress. For instance, they may request me to help with a haircut, but after 10 or 15 minutes, they would say, “I'm dizzy now. I don't want to see you now. I want to stay alone.” (N20)
			One day during the night shift, you might have a heart-to-heart talk with the patient. They would hold your hands and say, “Thank you for giving me strength and warmth.” Then the next day, the patient doesn't recognize or remember you, or they even start to resist you. You don't know the reasons for it, which is very frustrating. (N23)
		Lack of standards in spiritual care	Individuals have varied spiritual needs, so there is no single correct answer or set procedures guiding you in providing spiritual care. It [spiritual care] changes all the time. Therefore, it's quite stressful. (N5) We need to fulfill patients' emotional or spiritual needs before their deaths. I don't know how to take care of those patients or flow into their hearts. This can be stressful. (N8)
	Family needs	Futile resuscitations	Not every patient in our units can leave peacefully because some families persistently ask for resuscitations, possibly influenced by China's traditional values. These families believe that they must resuscitate their parents to show filial piety and care. I find it painful to witness these resuscitations for terminal patients, but we must comply in most situations. That's what the family asks for, and we must follow. (N1) Many patient families consent to hospice care, but when the patients are close to death, their families regret, change their minds, and force you to intervene. The families can't bear the judgments or infamies from society, so they ask for resuscitations and continue letting the elders live with pain and suffering. This situation is very stressful, but I can't make any changes. (N7)
		Disease concealment	I believe that every patient family who conceals illness conditions from the patients will eventually regret it. While I would suggest not concealing such information, I respect the family's decisions, and I have no right to interfere. Therefore, I will be more careful in handling these situations and feel stressed in the meantime. (N8) It's very common for patient families to request that we conceal the real conditions of patients, not informing them about the limited time they have left. I find it challenging and distressing to provide hospice care while concealing. (N19)
	Personal burdens	Lack of knowledge and skills	There are situations where I find myself unsure of how to handle them, possibly due to a lack of capabilities and techniques. I feel that I haven't learned enough or don't possess sufficient knowledge, and this can be quite stressful. (N5) Hospice care has high requirements for nurses, not only in terms of working experience but also in terms of personal insights and approaches to handling problems. I often find that my knowledge and techniques are still insufficient, which can be quite stressful when facing certain issues. (N12)
		Struggle of emotional boundaries	I think that our job is kind of cruel. You need to build emotional relationships with patients, but every relationship has the same outcome—they will pass away. Therefore, I think it's very cruel and very sad for us, hospice nurses. This requires a strong psychological state. Every time I think of their leaves, I feel deeply sorrowful. (N11) I take care of them, listening to music together, going for walks, and investing both effort and emotions. We then build strong emotional relationships. When they pass away, it feels akin to losing a family member. It's a deeply sorrowful experience. You know that they will have a such end, but you can not control your tears. (N23)
		Death anxiety	I believe that working in the hospice unit can be challenging because, at times, emotions are difficult to control. After witnessing patients' deaths, I occasionally find myself losing hope in life and feeling that things are meaningless. (N14) I contemplate my own mortality, acknowledging that one day I will experience the same fate. I also think about the eventual loss of my family members. There is no solution; I must accept it. Every time these thoughts raising, I feel hurtful. (N26) Only a few people will witness a living human being disappear in front of their eyes, and we, as hospice workers, experience this regularly, without any psychological preparation, at anytime and anywhere. All I can say is that it is cruel. Sometimes you would feel that life is nihilistic. What's the meaning of human existence? (N30)

Organizational level	Organizational constraints	Insufficient financial support and human resources	I feel stressful every day. The government provides minimal funding, and we receive low pay. Why am I still dedicated to this job? This is a crucial question many people are facing. The most basic reality is that no matter how passionate you are about hospice care, you must survive. While money may not be your top priority, you need to ensure, at the very least, your daily necessities. (N7) Working here, there is an imbalance between your effort and your pay. In our hospital, as hospice nurses, we are the busiest but receive very low pay. (N16) Personnel are very lacking. Many hospice nurse specialists are not specially providing hospice care. Most of them must take on responsibilities in other wards and provide hospice care during their free time. So, this is very challenging. In fact, all our cases involving spending our own time and money to care for patients. (N8) Actually, hospice care is an additional responsibility. While tending to patients in the hospice wards, there are other patients you need to take care of as well. Initially, we didn't have the responsibility of hospice care, but now we have extra specialized tasks due to the addition of hospice wards. I currently handle it with my devotion. (N23)
		Negative leadership behaviors	The head nurse frequently criticizes and scold us in the morning meetings. Working in a department where death is a common occurrence is already quite stressful. Having to face criticism early in the morning adds to the stress throughout the day. I find this situation very frustrating. (N2) I feel like the head nurse doesn't really care about their duties. However, when their supervisor asks for task outcomes, they turn to you and request information. What can you do? You can't say it's not your responsibility. They never specify what I should do and what I don't need to do, which is quite annoying. They seem unwilling to take responsibility but frequently ask you to handle tasks. (N16)
		Conflict philosophies in healthcare	I think there is another major stressor. Hospice care has only been practiced well by nurses but not by doctors. The medical philosophies are mismatched. Many doctors have not recognized hospice care, and this is disadvantageous to cooperate as a team. (N16) Nurses have a higher passion for hospice care. But as a nurse, your voice is often not heard. After all, doctors have all the rights to decide how to treat patients, and they are seen as having a higher status by patients. Nurses face many stresses during their practices, especially when doctors don't recognize your work. (N19)

Societal level	Social values	Avoid conversations about death	China has a long history of Confucianism for thousands of years. Due to its influence, we only discuss births and lives but not deaths. Many people still avoid discussing or facing death; thus, the death education is very challenging. People generally have a low acceptance toward it. Hospice care is about discussing death, but no one is willing to engage in conversations about death. Therefore, this job is quite stressful. (N3) When we tried to promote hospice care to the patient, they did not accept it very well due to the impact of our Chinese traditional cultures. They would say that death bring bad luck, then they always avoid any conversations about it. People always misunderstand about hospice care, and this misunderstanding make you feel stressful. (N23)
		Pragmatism	We provide some comforting services to the patients, such as meditation and aromatherapy, but they perceive these as ineffective for their illness. You meticulously prepare those services, but it doesn't come with positive feedback, then you would feel stressed. I even started to question the purpose of this job. (N6) They [patient families] don't care about it [comforting care] because they believe that it's not necessary or beneficial for their parents [the patients]. Comforting care is perceived as lacking practicality. I think this is detrimental to our work and feel a little frustrated. (N13)
		Implicit communication mode	The implicit expression of Chinese people makes it hard for them to express their emotions. It's very difficult for you to let them speak out. Hospice care encourage patients to reflect on their emotions and feelings, but we just can't do it. It's hopeless, and I don't know how to do it. (N2) Chinese people are often reserved, and many of them may not be adept at expressing themselves or do so in an ambiguous way. So, we can't have some deep conversations, and this could be another stress in my work. (N21)

Acute stressors	Unexpected events	Patient suicide	We have a patient committed suicide during the night, without a single sign. You can't figure out why this happened. After that, I become very concerned about the patients, especially about their psychological problems. (N6)
		Sudden patient death	One older patient suddenly passed away. I made rounds of the ward, and the nursing assistant did as well, but the patient suddenly died without warning. During our meeting, the head nurse and doctors questioned me if I did the scheduled word inspections and if I checked their breaths. I just stood there and started self-doubting. It's very stressful. (N11) Patients could pass away peacefully in their sleep, with stopped heartbeats and no breaths, even if they were not in a severe condition. When patient families touched their bodies, they found them cold. Overwhelmed with grief, they rushed to the nurse station, crying and eventually breaking down. You can imagine how stressful it was for us (N16)

## Data Availability

Due to the ethical obligations of the authors, the data used in this study are not available.

## Supplementary Materials

Supplementary Materials Supplementary Material 1: Research Opportunities Inquiry at Your Institution.
