# Supplementary material for: “Negative Energy Magnetic Field”: A Descriptive Qualitative Study on Occupational Stressors among Chinese Hospice Nurses
Source: J Nurs Manag. 2024 Aug 29;2024:3311735. doi: 10.1155/2024/3311735 (PMC11918853; doi:10.1155/2024/3311735)
Supplement: Supplementary Materials — Supplementary Material 1: Research Opportunities Inquiry at Your Institution. [file 3311735.f1.docx]

To whom it may concern,

My name is Yanming Wu, and I am a Ph.D. student at the Nursing School of Shanxi Medical University. I am conducting research under the supervision of Professor Hui Yang, focusing on exploring the stress experiences of hospice care nurses during care-providing processes.

In recent years, your institution has accomplished remarkable achievements and accumulated valuable experience in the development of hospice care. To learn about the advanced experiences and practices in hospice care at your institution, we would like to conduct on-site research for approximately 2 weeks, interviewing 5-6 nurses who have been engaged in hospice care for 1 year or more. This research study aims to understand nurses' care-providing experiences in clinical settings through interviews, with a primary focus on their stress experiences while providing care to terminal patients. I would greatly appreciate your support and assistance in this endeavor.

Best regards,

Yanming Wu

Ph.D. Student

Nursing School of Shanxi Medical University
